# What calls for service tell us about suicide: A 7-year spatio-temporal analysis of neighborhood correlates of suicide-related calls

**DOI:** 10.1038/s41598-018-25268-0

**Published:** 2018-04-30

**Authors:** Miriam Marco, Enrique Gracia, Antonio López-Quílez, Marisol Lila

**Affiliations:** 1 Department of Social Psychology, University of Valencia, Valencia, 46010 Spain; 2 Department of Statistics and Operations Research, University of Valencia, Valencia, 46100 Spain

## Abstract

Previous research has shown that neighborhood-level variables such as social deprivation, social fragmentation or rurality are related to suicide risk, but most of these studies have been conducted in the U.S. or northern European countries. The aim of this study was to analyze the spatio-temporal distribution of suicide in a southern European city (Valencia, Spain), and determine whether this distribution was related to a set of neighborhood-level characteristics. We used suicide-related calls for service as an indicator of suicide cases (n = 6,537), and analyzed the relationship of the outcome variable with several neighborhood-level variables: economic status, education level, population density, residential instability, one-person households, immigrant concentration, and population aging. A Bayesian autoregressive model was used to study the spatio-temporal distribution at the census block group level for a 7-year period (2010–2016). Results showed that neighborhoods with lower levels of education and population density, and higher levels of residential instability, one-person households, and an aging population had higher levels of suicide-related calls for service. Immigrant concentration and economic status did not make a relevant contribution to the model. These results could help to develop better-targeted community-level suicide prevention strategies.

## Introduction

Suicide is a major social and public health problem worldwide^1^. In 2012, there were over 800,000 suicide deaths around the world^2^. Europe is the WHO region with the highest rates of suicide worldwide, with 14.1 per 100,000 inhabitants, followed by the European Union, where the rates are around 11 per 100,000^3^. In Spain, where this study was conducted, and according to 2015 data, suicide was the first leading external cause of death, 1.9 times more than road traffic injuries, and 12.6 times more than homicides^4^. In the same year, 3,602 people died by suicide, which represents almost 10 suicides a day^5^. The problem is even greater if we take into account parasuicide (i.e., suicide attempts), which cannot be easily measured. Despite the social and economic costs of suicide in our societies, there is still a lack of prevention strategies that can adequately deal with this phenomenon^6,7^. Focusing on the range of variables that could explain suicidal behavior from different levels of analysis may help to design better-informed preventive actions.

Recently, a growing number of studies have suggested that neighborhood-level variables have an impact on suicide risk beyond individual-level factors^8–11^. Starting from the research of Congdon^12^, these studies point to the link between suicide risk and three sets of factors: social deprivation, social fragmentation, and rurality^8,9,13,14^. Neighborhood social deprivation, measured by different indicators such as poverty rate, unemployment, occupational social class, and education level, has been positively linked with suicide in a number of studies^9,14–16^. Neighborhood social fragmentation also has shown a positive relationship with suicide risk^9,14^. Indicators such as high levels of residential instability, high percentage of one-person households, and high divorce rates have been related to higher risks of suicide behavior, even after controlling for social deprivation^10,17^. Another stream of research studies has compared rural and urban areas in suicide risk, suggesting that the risk of suicide is higher in rural areas^18,19^. Research using population density as a proxy of rurality has also shown that areas with higher population density have lower risks of suicide^9,20^.

In addition to these variables, other neighborhood-level indicators have also been explored as predictors of suicide risk. For example, some research has focused on the relationship between ethnic density and suicide rates^18,21,22^. This relationship, however, is still inconclusive. Although some studies have found higher suicide rates in areas with high density of ethnic minorities^21–23^, other found no such significant relationship^18^.

Finally, although age has been positively linked to suicide risk at the individual level—i.e., higher suicide rates among older people^6,8,24^—the influence of this variable at the neighborhood-level has received less research attention. Some research suggests, however, that neighborhoods with higher levels of population aging (i.e., the ratio of elderly to young populations), tend to show higher suicide rates^15^.

## Present Study

The aim of this study was to analyze the spatio-temporal distribution of suicide-related calls for service in a southern European city (Valencia, Spain), and whether this distribution was related to a set of neighborhood-level characteristics: economic status, education level, residential instability, one-person households, population density, immigrant concentration, and population aging. We used suicide-related calls for service as an indicator of suicidal behavior. This measure captures all calls for service received by the police related to suicide and parasuicide interventions, and has previously been considered as an indicator of suicidal behavior^25^.

A Bayesian spatio-temporal approach was used to deal with the methodological biases usually present in ecological studies such as overdispersion or spatial autocorrelation^26,27^. This methodological approach is common in public health analysis^26–28^ and is increasingly being used in the geographical study of suicide^14,15,29–36^.

Previous research has shown that high-resolution studies taking a small-area approach are more appropriate than other levels of aggregation with lower resolution (such as districts, counties or cities) for assessing spatial variations and neighborhood influences on suicide risk^14,37^. Similarly, they have shown the importance of taking into account a temporal approach when analyzing social problems in the neighborhood. More specifically, studies on suicide have shown the relevance of a temporal perspective, which could improve our knowledge of this outcome^37^. A previous study analyzed the baseline distribution of suicide-related calls comparing different spatial and spatio-temporal models and it showed that using a spatio-temporal approach improved the results obtained with a purely spatial model^37^. Research has also shown that yearly studies could mask the effect of seasonality found in suicide events^1,37,38^, and therefore using shorter temporal periods, such as seasons, is more appropriate^37^. Thus, we chose a spatio-temporal approach for this study.

This study therefore analyzes the influence of a set of neighborhood-level characteristics on the spatio-temporal distribution of suicide calls for service using census block groups (the smallest area available), and trimesters as the temporal unit in a Southern European city. To the best of our knowledge, this is the first study that has used a Bayesian spatio-temporal modeling approach to analyze neighborhood influences on the spatio-temporal distribution of suicide-related calls in a Southern European city.

## Methods

### Study area and time

Valencia is the third largest city in Spain, with a population of 790,201 inhabitants (2016 data)^39^. The census block group—the smallest administrative unit available—was used as the proxy for the neighborhood. The city of Valencia has 552 census block groups (population range 630–2,845).

The number of suicide-related calls for service was used as the outcome variable. Valencia Police Department provided data of all suicide-related calls requiring police intervention in the city of Valencia from 2010 to 2016. There were 6,537 calls in this period, of which 142 were related to suicide deaths, and 6,395 to suicide attempts. The address where the incident leading to a police intervention occurred was geocoded and located on the map of Valencia. Each year count was divided in 3-month periods to assess seasonality (period 1 = January to March, period 2 = April to June, period 3 = July to September, and period 4 = October to December). Thus, the outcome variable was divided into 28 periods.

### Covariates

Several indicators were collected for each census block group. In line with previous studies, indicators of social deprivation, social fragmentation and population density were assessed^9,14^, as well as the variables population aging and immigrant concentration. Data was collected from the official records of the statistics office of Valencia City Hall corresponding to the year 2013. These indicators did not present significant differences across the years of the study, so the central year was selected.

#### Social deprivation indicators

Two variables were used as social deprivation indicators: economic status and education level. For economic status an index was constructed through an unrotated factor analysis using several highly correlated economic indicators, including cadastral value, percentage of high-end cars, percentage of financial businesses (number of businesses related to finances and insurances activities by the total of businesses), and percentage of commercial businesses (number of businesses related to commercial activities by the total of businesses). For the second variable, the average education level of neighborhood residents was measured on a 4-point scale, where 1 = less than primary education, 2 = primary education, 3 = secondary education, and 4 = college education.

#### Social fragmentation

We used two indicators to measure social fragmentation: residential instability and one-person households. The residential instability variable was calculated as the proportion of the population that had moved into or out of each census block group during the previous year (rate per 1,000 inhabitants). One-person households were measured as the number of households with only one person per total number of households.

#### Population density

Population density per square kilometer was used as a proxy of rurality.

#### Population aging index

An index of the population aging (i.e., the ratio of elderly to youth populations) was measured as the number of people aged 65 years or over per hundred people under the age of 15 years old.

#### Immigrant concentration

This was measured as the percentage of immigrant population in each census block group.

#### Seasonality

To explore the effect of seasonality, a dummy variable was introduced creating three binary variables that account for the first three trimesters; the fourth trimester was selected as the reference.

Table 1 shows the descriptive statistics for all the variables.

**Table 1:** Variables (Mean, Standard Deviation, Minimum and Maximum Values) at the Census Block Group and Year Level (2013 data).

Variable	Mean (SD)	Min	Max
Deprivation			
Economic status			
Property values (€)	260.10 (74.61)	111.50	590.70
High-end cars (%)	5.75 (3.62)	1.30	24.80
Commercial businesses (%)	34.03 (9.21)	7.50	66.40
Financial businesses (%)	18.15 (7.77)	0	43.20
Education level	3.15 (0.33)	2.39	3.86
Population Density	3,346 (1,736.94)	107	13,112
Fragmentation			
Residential instability	268.00 (87.98)	91.10	649.80
One-person households	32.72 (6.58)	15.46	54.78
Aging index	151.20 (60.00)	16.20	501.10
Immigrant concentration (%)	13.28 (6.53)	1.90	40.20
Suicide-related calls	0.26 (0.57)	0	7

Abbreviations: SD, standard deviation; Min, minimum; Max, maximum.

### Data analysis

A conditionally independent Poisson distribution was used to model the number of suicide-related calls for service. Specifically, the outcome in each census block group and each period was expressed as follows:

1 $${O}_{it} \sim Po({E}_{it}\exp ({\eta }_{it})),\,\,i=1,\,\ldots ,\,552,\,\,t=1,\,\ldots ,\,28$$

where ${E}_{it}$ is a fixed quantity representing the expected number of calls in census block group $i$ during period *t* in proportion to the population in Valencia in this census block group. ${\eta }_{it}$ is the log relative risk for every area and period.

Two different models were used. Both models included a spatio-temporal effect via the ${\eta }_{it}$. We followed an autoregressive approach, which combines autoregressive time series and spatial modeling using a spatio-temporal structure where relative risks are both spatially and temporally dependent^40^. The log relative risk for the first period was defined as:

2 $${\eta }_{i1}=\mu +{\alpha }_{1}+{(1-{\rho }^{2})}^{-1/2}\cdot ({\varphi }_{i1}+{\theta }_{i1})$$

while the relative risks for the following periods were defined as:

3 $${\eta }_{it}=\mu +{\alpha }_{t}+{\beta }_{q(t)}+\rho ({\eta }_{i(t-1)}-\mu -{\alpha }_{t-1})+{\varphi }_{it}+{\theta }_{it})$$

where $\mu$ is the intercept, $\alpha$ is the mean deviation of the risk in the period *t*, $\rho$ represents the temporal correlation between the spatial effects of each period, and ${\varphi }_{{\rm{it}}}$ and ${\theta }_{{\rm{it}}}$ refer to structured and unstructured spatial random effects, respectively.

We incorporated a ${\beta }_{q(t)}$, which represents the mean deviation of the risk in trimester $\,q(t)$. The fourth trimester was selected as the reference, and the other three trimesters were compared to it.

The first model only accounted for this spatial-temporal effect and included seasonality as a covariate, while the second model incorporated different covariates to analyze the influence of neighborhood-level characteristics in the outcome. Seven covariates were introduced to this second model: economic status, education level, residential instability, one-person households, population density, population aging index, and immigrant concentration. The final model was as follows:

4 $${\eta }_{it}=\mu +{\alpha }_{t}+{X}_{i}\beta +\rho ({\eta }_{i(t-1)}-\mu -{\alpha }_{t-1})+{\varphi }_{it}+{\theta }_{it})$$

where ${X}_{i}$ is the vector of covariates, and $\beta$ is the vector of regression coefficients.

A Bayesian approach was followed for both models. Accordingly, appropriate prior distributions were assigned for the parameters, namely, vague Gaussian distributions for the fixed effects $\beta$; $\mu$ was specified as an improper uniform distribution; the autoregressive term $\rho$ was modeled as a uniform over the whole space $U(\,-\,1,1)$; and the structured effect was specified by a conditional spatial autoregressive (CAR) model^41^:

5 $${\varphi }_{i}|{\varphi }_{-i} \sim N(\frac{1}{{n}_{i}}{\sum }_{j \sim i}{\varphi }_{j},\frac{{\sigma }_{\varphi }^{2}}{{n}_{i}})$$

where ${n}_{i}$ represents the number of neighboring areas of each census block group $i$, ${\varphi }_{-i}$ indicates the values of the $\varphi$ vector except the component $i$, ${\sigma }_{\varphi }$ is the standard deviation parameter, and $j \sim i$ is the units $j$ neighbors of census block group $i$. The unstructured spatial effect $\,\theta$ was modeled by means of independent identically distributed Gaussian random variables $N(0,{{\sigma }_{\theta }}^{2})$. Finally, uniform distributions were used for the three hyperparameters ${\sigma }_{\alpha },\,{\sigma }_{\varphi },\,{\sigma }_{\theta }\, \sim \,U(0,1)$, following the structure of the hierarchical Bayesian models.

In order to test the robustness, a sensitivity analysis was conducted using different prior distributions for the hyperparameters. The posterior distributions showed consistent results (see Supplementary Table S1).

To implement the models, simulation techniques based on Markov Chain Monte Carlo (MCMC) were used with the software R^42^ and the R2WinBUGS package. Three chains with 50,000 iterations were generated, and the first 10,000 were discarded as burn-in. The deviance information criterion (DIC) was used to compare models and select the best fit: the smaller the DIC, the better the fit^43^.

## Results

The results of the two Bayesian autoregressive models are presented in Table 2.

**Table 2:** Mean, standard deviation and 95% credible interval of the parameters of the autoregressive models.

	Model 1 (Autoregressive model without covariates)				Model 2 (Autoregressive model with covariates)			
	Mean	SD	95% CrI		Mean	SD	95% CrI	
Intercept	−0.362	0.045	−0.450	−0.275	−0.136	0.528	−1.265	0.767
Economic status					−0.029	0.057	−0.141	0.082
Education level					−0.269	0.160	−0.645	−0.021
Density					−0.015	0.002	−0.018	−0.012
Residential instability					0.001	0.001	0.000	0.002
One-person households					0.002	0.000	0.002	0.003
Aging					0.002	0.000	0.001	0.002
Immigrant concentration					−0.007	0.008	−0.024	0.009
Trimester 1	−0.122	0.057	−0.230	−0.008	−0.119	0.053	−0.220	−0.014
Trimester 2	0.093	0.059	−0.024	0.208	0.093	0.058	−0.019	0.206
Trimester 3	0.118	0.053	0.016	0.227	0.119	0.054	0.011	0.222
${{\rm{\sigma }}}_{\theta }$	0.359	0.019	0.323	0.398	0.377	0.021	0.337	0.416
${{\rm{\sigma }}}_{\varphi }$	0.160	0.030	0.102	0.220	0.144	0.080	0.011	0.256
${\sigma }_{\alpha }$	0.106	0.032	0.051	0.178	0.106	0.030	0.055	0.166
ρ	0.903	0.009	0.885	0.919	0.881	0.011	0.859	0.900
DIC	24,577					24,546.7		

Abbreviations: SD, standard deviation; CrI, credible interval; σ_*θ*_, standard deviation spatially unstructured term; σ_*ϕ*_ standard deviation structured term; σ_∝_, standard mean deviation of the risk; *ρ* temporal correlation.

Model 1 represents the autoregressive model without covariates, while Model 2 incorporates the effect of the covariates of the study. Note that Model 2 showed a relevant decrease in the DIC (30.3 units of decrease), and was therefore selected as the final model.

In this final model, indicators of social deprivation (education level), social fragmentation (residential instability and one-person households), and population density were associated with higher levels of suicide-related calls for service. Population aging was also relevant to the model. All these variables showed a posterior probability of being over or under zero higher than 95% (i.e., their 95% credible intervals did not include zero). Economic status and immigrant concentration, however, did not show a relevant relationship with the outcome, including zero in the 95% credible interval. These results indicate that areas with lower education level, lower levels of population density, higher residential instability and concentration of one-person households, and higher population aging have higher levels of suicide-related calls for service.

The seasonality effect was also relevant. Specifically, results show that the estimated number of suicide-related calls increases in the second and the third trimesters (i.e., from April and September), with a higher increase in the third trimester. In contrast, in the first trimester the estimated number of suicide-related calls is lower. The first and third trimesters had a 95% credible interval where zero was not included. For the second trimester, although its 95% credible interval includes zero, the posterior probability of being positive was 0.948, so we considered its effect relevant.

**Figure 1:**
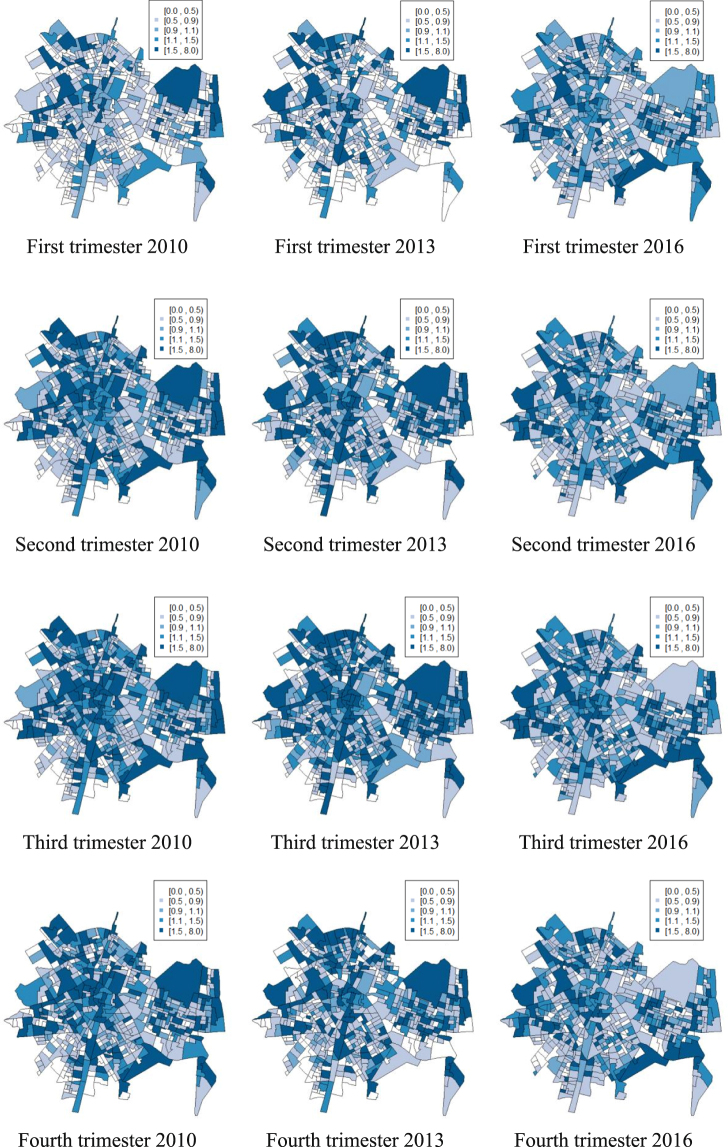
Relative risk for the four trimesters of 2010, 2013 and 2016 (maps created by the software R version 3.4.3., available in https://www.R-project.org).

The Bayesian autoregressive model used allows us to analyze both spatial and temporal trends. Figure 1 shows the relative risk for a sample of the periods: the four trimesters of 2010, 2013 and 2016 (see Supplementary Fig. S1 for all maps). This figure reflects the differences between census block groups in each year, where areas with relative risks greater than 1 indicate an above-average probability. The general pattern shows that the highest risks are concentrated in some areas of the center, the north-west and the east of the city. These risks are higher in the second and the third trimester than in the first and the fourth.

**Figure 2:**
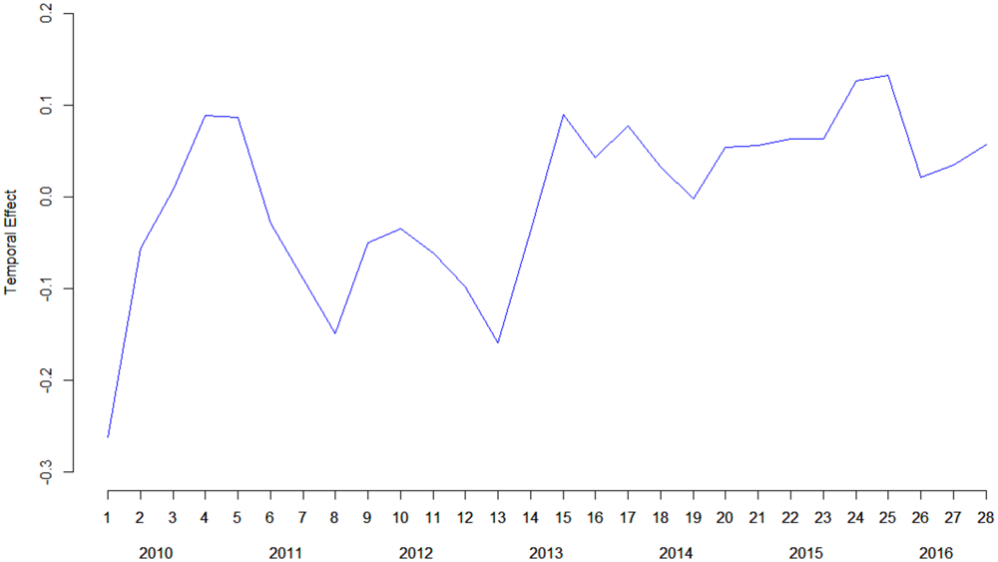
Temporal effect during the period (2010–2016).

Regarding the temporal pattern, the parameter $\rho$ had a value of 0.88, indicating a high temporal correlation between a particular trimester and its predecessor. Figure 2 shows the temporal effect for each trimester. Despite the increasing trend over the years (there is no evidence of stabilization), this increase was not constant, but we found some clear peaks within the year as a result of the seasonality effect. Therefore, it is important to take into account seasonality to better capture the temporal trend of suicide-related calls for service.

## Discussion

This study explored the influence of neighborhood-level characteristics on the spatio-temporal distribution of suicide-related calls for service in Valencia, Spain. An autoregressive model following a Bayesian approach was conducted to assess both spatial and temporal effects. Although previous studies have shown the relationship between ecological variables and the risk of suicide^11,14,30,33^, to the best of our knowledge there are no studies assessing the geography of suicide in South European countries using a small area approach^44^.

Our results showed that neighborhoods with lower levels of education and population density, and higher levels of residential instability, higher concentration of one-person households, and higher population aging had higher levels of suicide-related calls for service. These results are in line with previous research suggesting that social deprivation, social fragmentation, and low population density are closely related to suicide risk at the community level^8–11,13,15,45^.

Economic status and immigrant population did not make a relevant contribution to the model. Previous studies have found a negative relationship between economic status and suicide-related outcomes^9,10,14,30^. The use of different measures of economic status may explain these differences, as these studies usually included variables such as income and unemployment, which were not available for the present study. In our case, education level was the deprivation variable with the greatest contribution to the model.

Regarding the results for immigrant concentration, it is important to take into account differences Spain has from other countries. Previous studies were mostly conducted in English-speaking and northern European countries, and usually focused on ethnic differences or indigenous people, where they found a positive association between suicide rates and percentage of ethnic minorities^9,21,23,46^. However, some cultural factors may influence the relationship between immigration and suicide rates. In Valencia, the largest immigrant group is from Latin American countries (34%). A shared dominant language in Spain and Latin America could facilitate social integration in the community more than in English speaking countries, and may be a protective factor against suicidal behavior. Future research would benefit from cross-cultural studies to analyze the differences among countries in the relationship between suicide and immigration rates.

Furthermore, we found a seasonality effect, with a peak of calls in the second (April to June) and third (July to September) trimesters, and a decrease in the other two. These results are similar to previous research suggesting that suicide rates increase in spring and summer^24,38,47,48^. These results indicate the importance of taking seasonality into account when conducting a temporal analysis of suicide trends. Our results also showed an important increase in the number of suicide-related calls for service from 2010. The results suggest that suicide-related calls for service increased over the study period (2010–2016), and that there was no stabilization by the end of 2016. Future studies would benefit from incorporating long-term data, including the following years, to further analyze the evolution of suicide trends.

This study has both strengths and limitations. Its strengths include its location in a southern European city, as noted above, where there is a lack of studies on suicide at the neighborhood level^37^. Our results suggest that, despite the differences between countries, some neighborhood-level characteristics associated with suicide in the U.S. and northern European cities can also explain suicide variations in a southern European city. This paper, thus, provides new evidence about the spatio-temporal distribution of suicide calls in southern European cities and the neighborhood-level characteristics that may be related to this distribution. However, it is important to be cautious as this study was conducted in one specific city. Future research would benefit from conducting studies with a similar approach in other South-Europe cities.

This study used census block groups, which is more appropriate for addressing biases associated to aggregate data^49^. Moreover, we used an autoregressive model following a Bayesian approach. The Bayesian autoregressive model has been found to perform better than other spatio-temporal models, and some studies have showed that it provides a slightly better fit in terms of DIC^50^. The Bayesian approach also has some advantages over the frequentist perspective. Bayesian models let researchers incorporate prior information, and also address issues such as overdispersion and spatial autocorrelation^26,27,31^. The Bayesian approach is increasingly being used to analyze social outcomes such as crime or violence^49,51–58^, and also to study suicide^31,32,37^.

Among the limitations, as we noted before, some commonly used variables such as income levels or unemployment were not available at the census block group level. Individual characteristics (such as the person’s gender or age) were also unavailable. Future research would benefit from analyzing the possible different spatial patterns according to the sex or the age of the person making the suicide-related call for service in Valencia. Furthermore, this study is based on calls for service, and other commonly used measures of suicidal behavior (such as hospitalizations or medical records) were not analyzed. In addition, although our results suggest an increase in suicide-related calls for service, the study period was limited and does not allow us to draw strong conclusions. Clearly, future studies need to incorporate longer periods of time in order to further analyze the temporal trend of suicide-related calls for service. Finally, despite the autoregressive model having a better performance than other models, it requires a high computation time due to the complexity of the model and the considerable time periods covered by this study^37,50^.

In conclusion, this study illustrates the relevance of a number of ecological variables in explaining suicide-related calls for service. Addressing these neighborhood risk factors and focusing on the high-risk areas with a higher increase in suicide-related calls for service could help to develop better targeted community-level suicide prevention strategies.

## Electronic supplementary material

Supplementary Material
