# Supplementary material for: What calls for service tell us about suicide: A 7-year spatio-temporal analysis of neighborhood correlates of suicide-related calls
Source: Sci Rep. 2018 Apr 30;8:6746. doi: 10.1038/s41598-018-25268-0 (PMC5928118; doi:10.1038/s41598-018-25268-0)
Supplement: Supplementary file 1 — Supplementary Material [file 41598_2018_25268_MOESM1_ESM.pdf]

What calls for service tell us about suicide: A 7-year spatio-temporal analysis of neighborhood correlates of suicide-related calls

Miriam Marco[1], Enrique Gracia[1], Antonio López-Quílez[2] & Marisol Lila[1]

[1]Department of Social Psychology, University of Valencia, Valencia, 46010, Spain.

[2]Department of Statistics and Operations Research, University of Valencia, Valencia, 46100, Spain. Correspondence and requests for materials should be addressed to M.M. (email: Miriam.Marco-Francisco@uv.es)

Supplementary Table S1. Sensitivity analysis for the final model

	**Model using $U(0,2)$, for σ_θ, σ_ϕ and $\sigma_\propto$**				**Model using Ga(0.01,0.01) for τ_θ , τ_ϕ and $\tau_\propto$**			
	**Mean**	**SD**	**95% CrI**		**Mean**	**SD**	**95% CrI**	
Intercept	-.412	.502	-1.50	.464	.115	.374	-.638	.749
Economic status	-.054	.056	-.171	.049	-.013	.048	-.112	.078
Education level	-.308	.123	-.534	-.089	-.256	.113	-.448	-.021
Density	-.015	.002	-.019	-.012	.015	.002	-.019	-.012
Residential instability	.001	.001	.000	.002	.001	.001	.000	.002
One-person households	.002	.000	.002	.003	.003	.001	.002	.004
Aging	.002	.001	.001	.002	.002	.001	.001	.002
Immigrant concentration	-.009	.008	-.025	.008	-.009	.008	-.026	.007
Trimester 1	-.123	.055	-.249	-.001	-.122	.056	-.231	-.008
Trimester 2	.089	.057	-.025	.202	.090	.058	-.020	.204
Trimester 3	.116	.051	.015	.217	.118	.054	.019	.230
σ_θ	.370	.020	.330	.410	.374	.027	.340	.411
σ_ϕ	.214	.037	.153	.288	.177	.059	.088	.296
$\sigma_\propto$	.106	.030	.052	.172	.113	.027	.068	.177
ρ	.880	.010	.861	.901	.880	.010	.859	.899
DIC	24545.7				24549.7			

Abbreviations: σ_θ, standard deviation spatially unstructured term; σ_ϕ, standard deviation structured term; $\sigma_\propto$, standard mean deviation of the risk; τ_θ, precision of spatially unstructured term; τ_ϕ, precision of spatially structured term; $\tau_\propto$, precision of risk; SD, standard deviation; CrI, credible interval; ρ temporal correlation

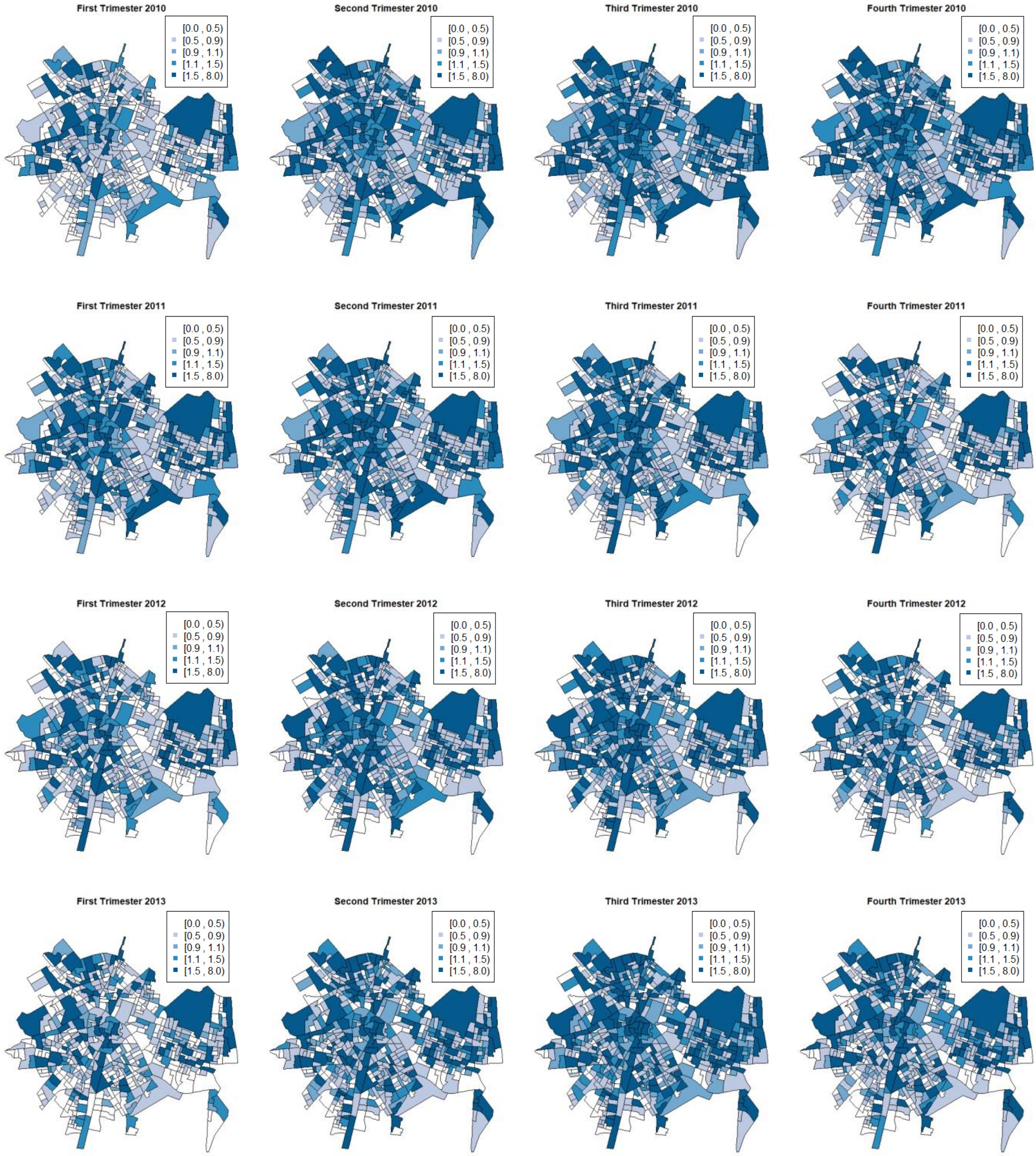
First Trimester 2010
[0.0 , 0.5)
[0.5 , 0.9)
[0.9 , 1.1)
[1.1 , 1.5)
[1.5 , 8.0)
Second Trimester 2010
[0.0 , 0.5)
[0.5 , 0.9)
[0.9 , 1.1)
[1.1 , 1.5)
[1.5 , 8.0)
Third Trimester 2010
[0.0 , 0.5)
[0.5 , 0.9)
[0.9 , 1.1)
[1.1 , 1.5)
[1.5 , 8.0)
Fourth Trimester 2010
[0.0 , 0.5)
[0.5 , 0.9)
[0.9 , 1.1)
[1.1 , 1.5)
[1.5 , 8.0)
First Trimester 2011
[0.0 , 0.5)
[0.5 , 0.9)
[0.9 , 1.1)
[1.1 , 1.5)
[1.5 , 8.0)
Second Trimester 2011
[0.0 , 0.5)
[0.5 , 0.9)
[0.9 , 1.1)
[1.1 , 1.5)
[1.5 , 8.0)
Third Trimester 2011
[0.0 , 0.5)
[0.5 , 0.9)
[0.9 , 1.1)
[1.1 , 1.5)
[1.5 , 8.0)
Fourth Trimester 2011
[0.0 , 0.5)
[0.5 , 0.9)
[0.9 , 1.1)
[1.1 , 1.5)
[1.5 , 8.0)
First Trimester 2012
[0.0 , 0.5)
[0.5 , 0.9)
[0.9 , 1.1)
[1.1 , 1.5)
[1.5 , 8.0)
Second Trimester 2012
[0.0 , 0.5)
[0.5 , 0.9)
[0.9 , 1.1)
[1.1 , 1.5)
[1.5 , 8.0)
Third Trimester 2012
[0.0 , 0.5)
[0.5 , 0.9)
[0.9 , 1.1)
[1.1 , 1.5)
[1.5 , 8.0)
Fourth Trimester 2012
[0.0 , 0.5)
[0.5 , 0.9)
[0.9 , 1.1)
[1.1 , 1.5)
[1.5 , 8.0)
First Trimester 2013
[0.0 , 0.5)
[0.5 , 0.9)
[0.9 , 1.1)
[1.1 , 1.5)
[1.5 , 8.0)
Second Trimester 2013
[0.0 , 0.5)
[0.5 , 0.9)
[0.9 , 1.1)
[1.1 , 1.5)
[1.5 , 8.0)
Third Trimester 2013
[0.0 , 0.5)
[0.5 , 0.9)
[0.9 , 1.1)
[1.1 , 1.5)
[1.5 , 8.0)
Fourth Trimester 2013
[0.0 , 0.5)
[0.5 , 0.9)
[0.9 , 1.1)
[1.1 , 1.5)
[1.5 , 8.0)

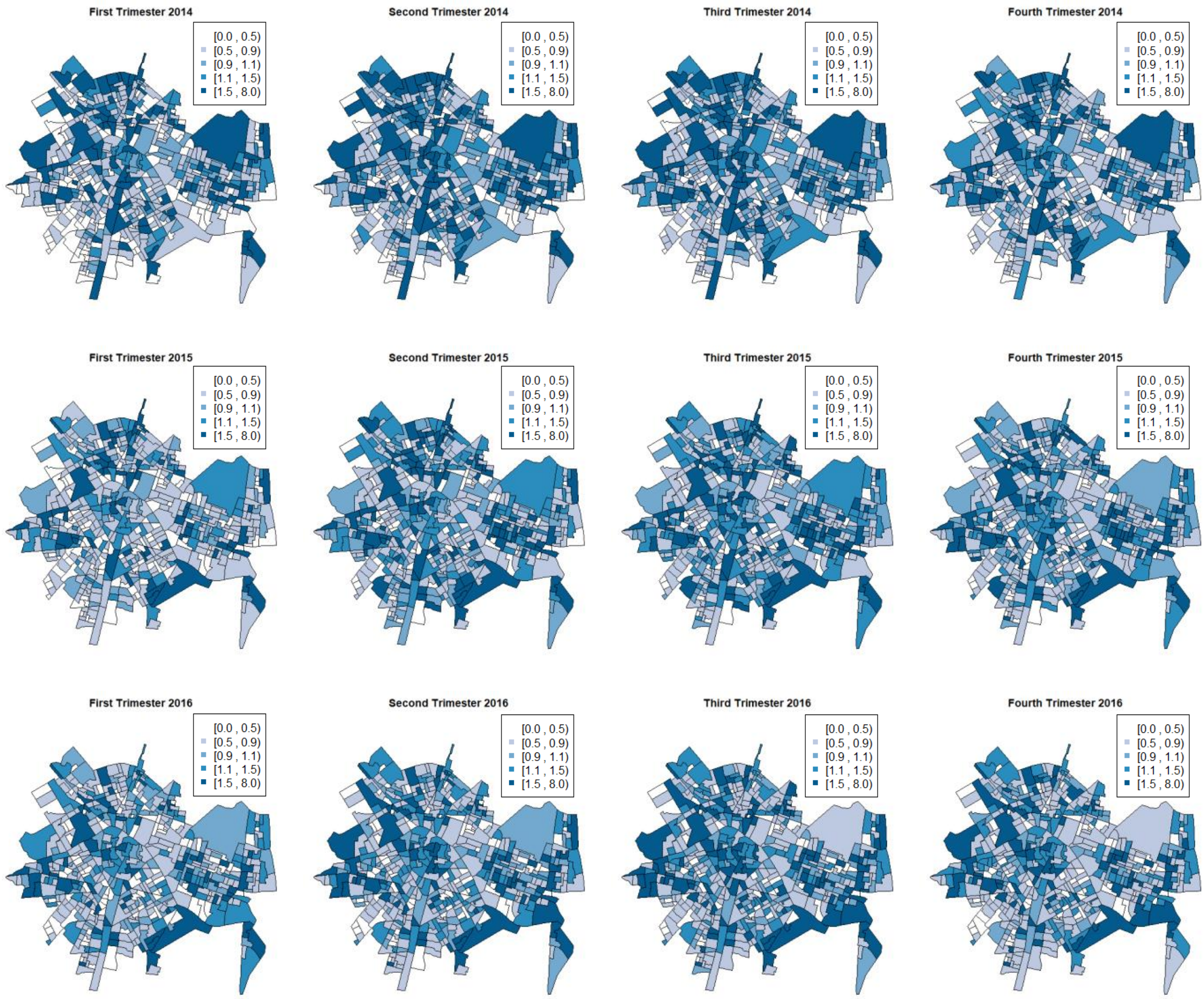

Supplementary Figure S1. Relative risk for all trimesters from 2010 to 2016 (maps created by the software R version 3.4.3., available in https:// R-project.org)
